# Supplementary figures and images for: Rational construction of controllable autoimmune diabetes model depicting clinical features
Source: PLoS One. 2022 Jan 21;17(1):e0260100. doi: 10.1371/journal.pone.0260100 (PMC8782301; doi:10.1371/journal.pone.0260100)

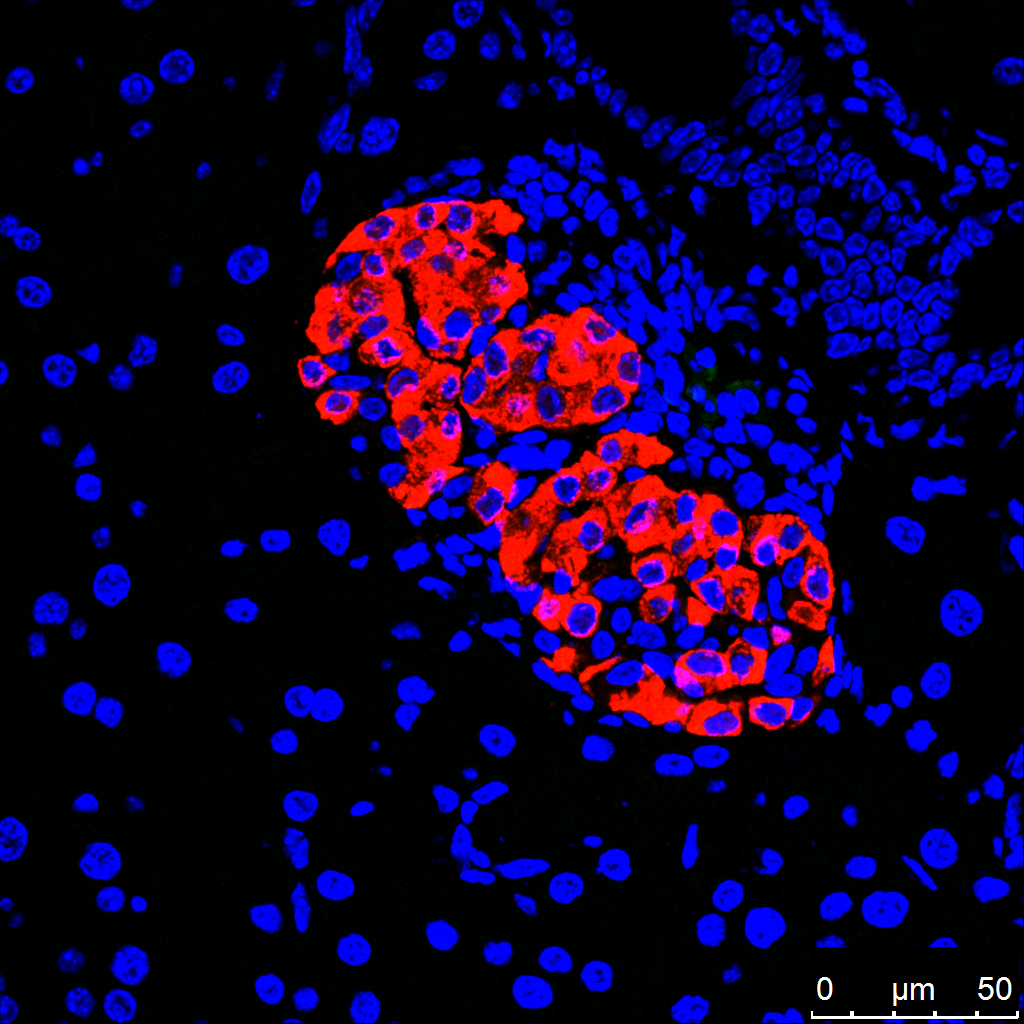

Supplement: S1 File — (ZIP) [file pone.0260100.s001.zip › PONE-D-21-15861R1-minimal data set-20211205yf/PONE-D-21-15861R1-Fig 1c-1d/Fig 1d NOD(17w).tif]

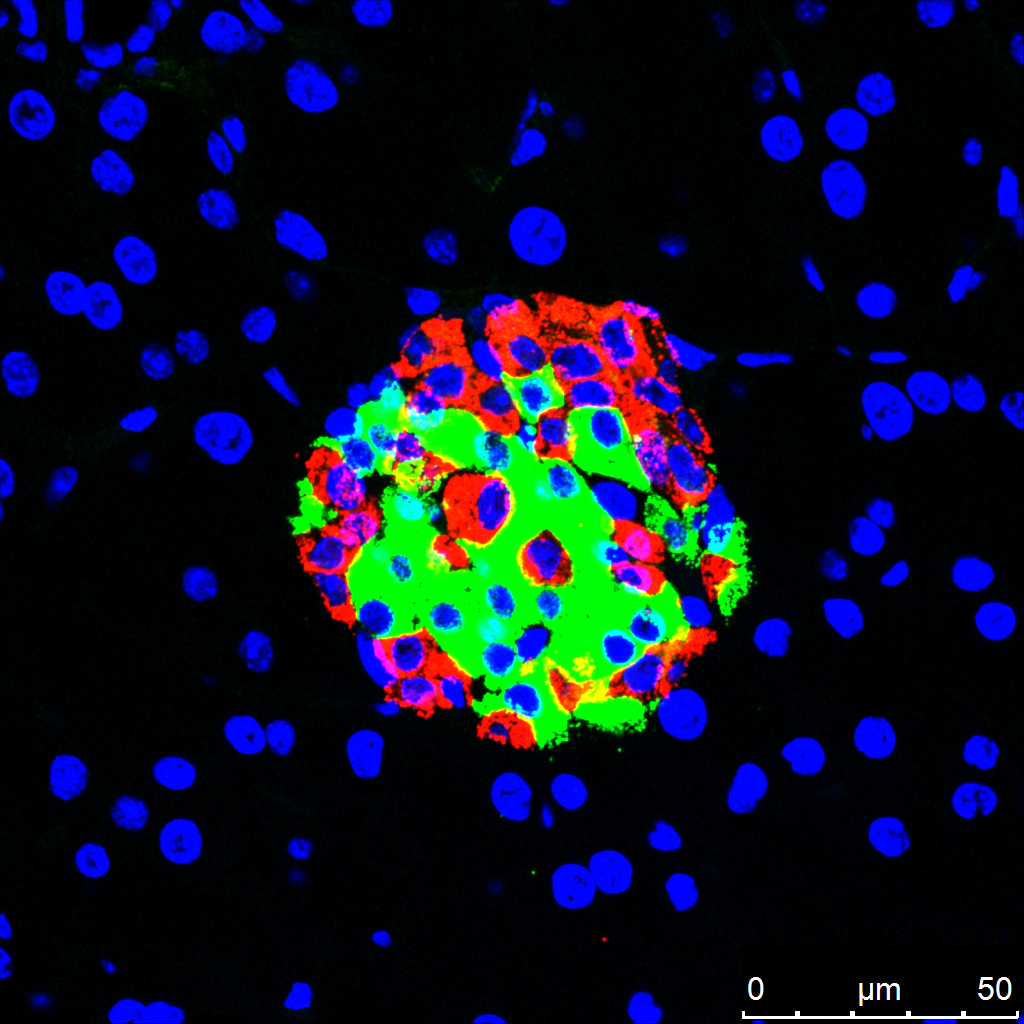

Supplement: S1 File — (ZIP) [file pone.0260100.s001.zip › PONE-D-21-15861R1-minimal data set-20211205yf/PONE-D-21-15861R1-Fig 1c-1d/Fig1c DT.tif]

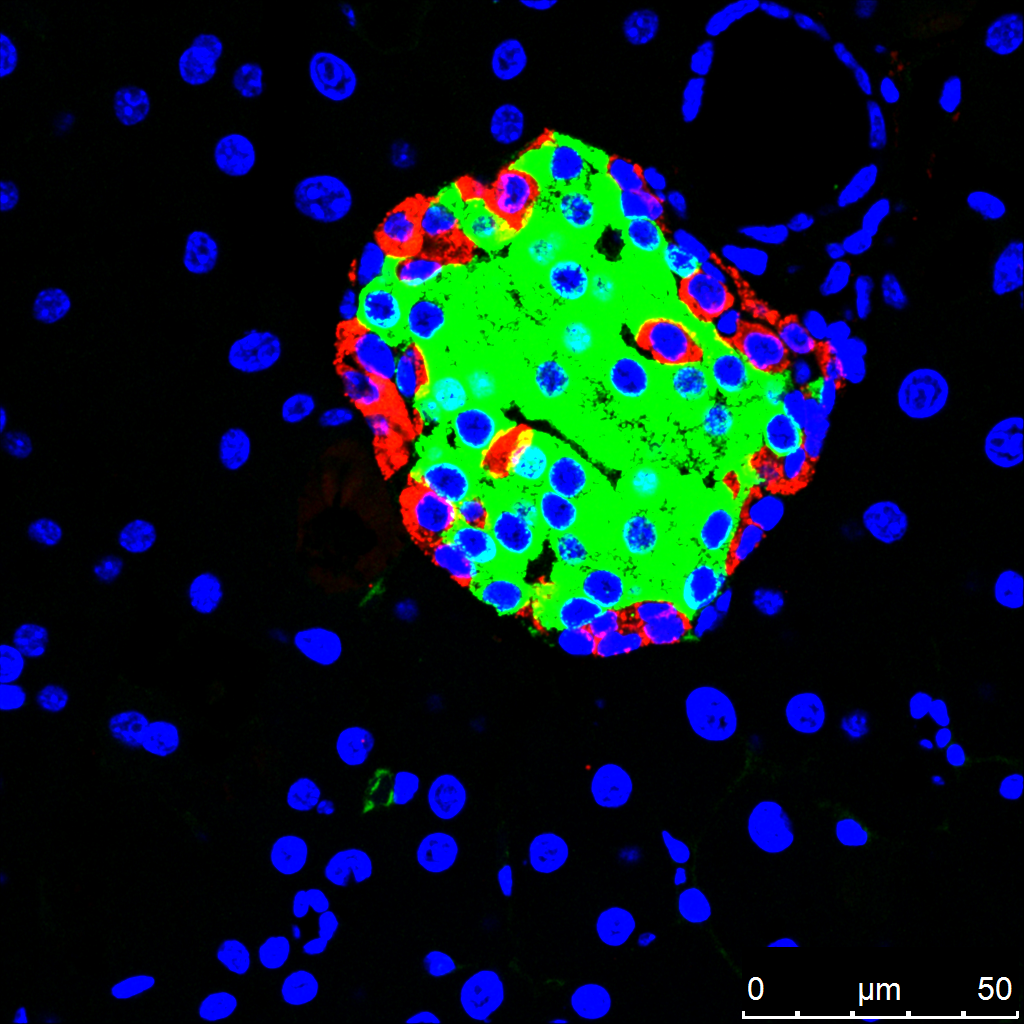

Supplement: S1 File — (ZIP) [file pone.0260100.s001.zip › PONE-D-21-15861R1-minimal data set-20211205yf/PONE-D-21-15861R1-Fig 1c-1d/Fig1c PBS.tif]

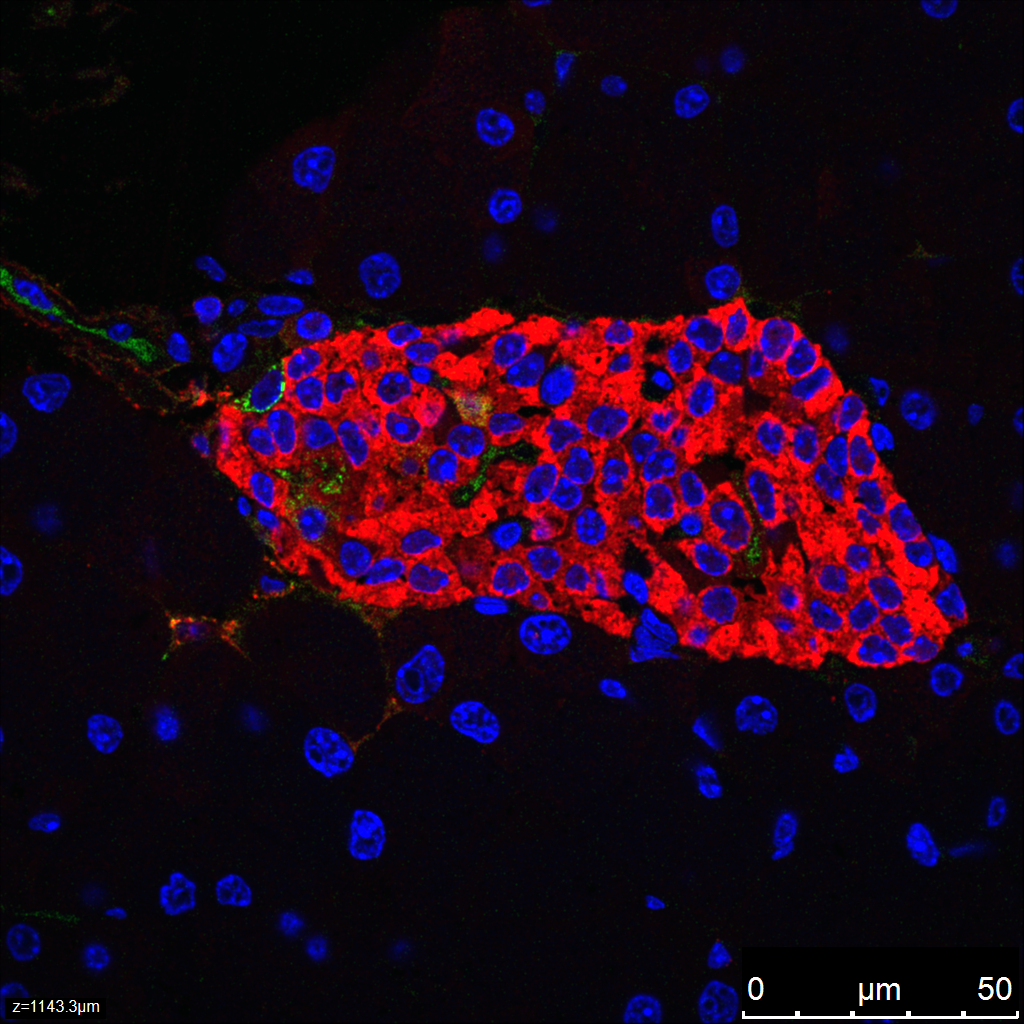

Supplement: S1 File — (ZIP) [file pone.0260100.s001.zip › PONE-D-21-15861R1-minimal data set-20211205yf/PONE-D-21-15861R1-Fig 1c-1d/Fig1c STZ+DT (Day 20).tif]

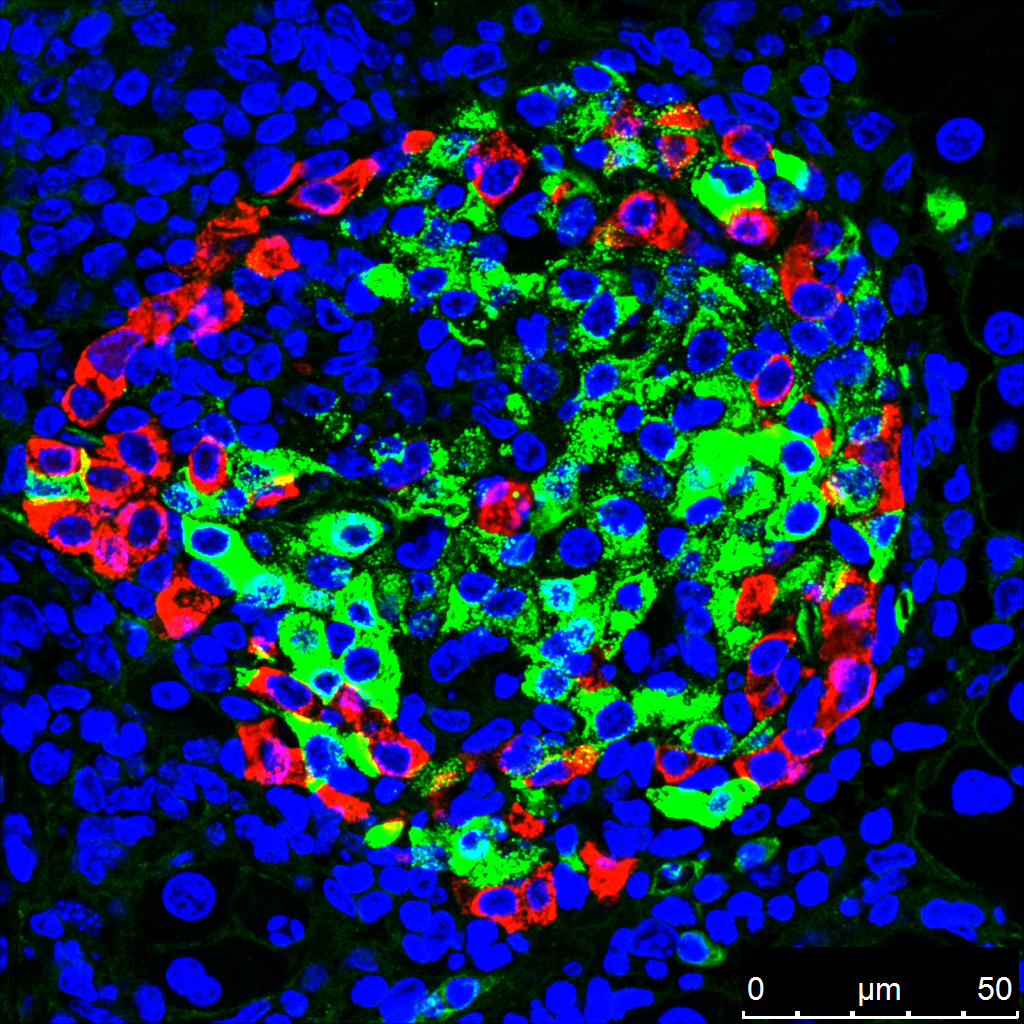

Supplement: S1 File — (ZIP) [file pone.0260100.s001.zip › PONE-D-21-15861R1-minimal data set-20211205yf/PONE-D-21-15861R1-Fig 1c-1d/Fig1c STZ+DT (Day 6).tif]

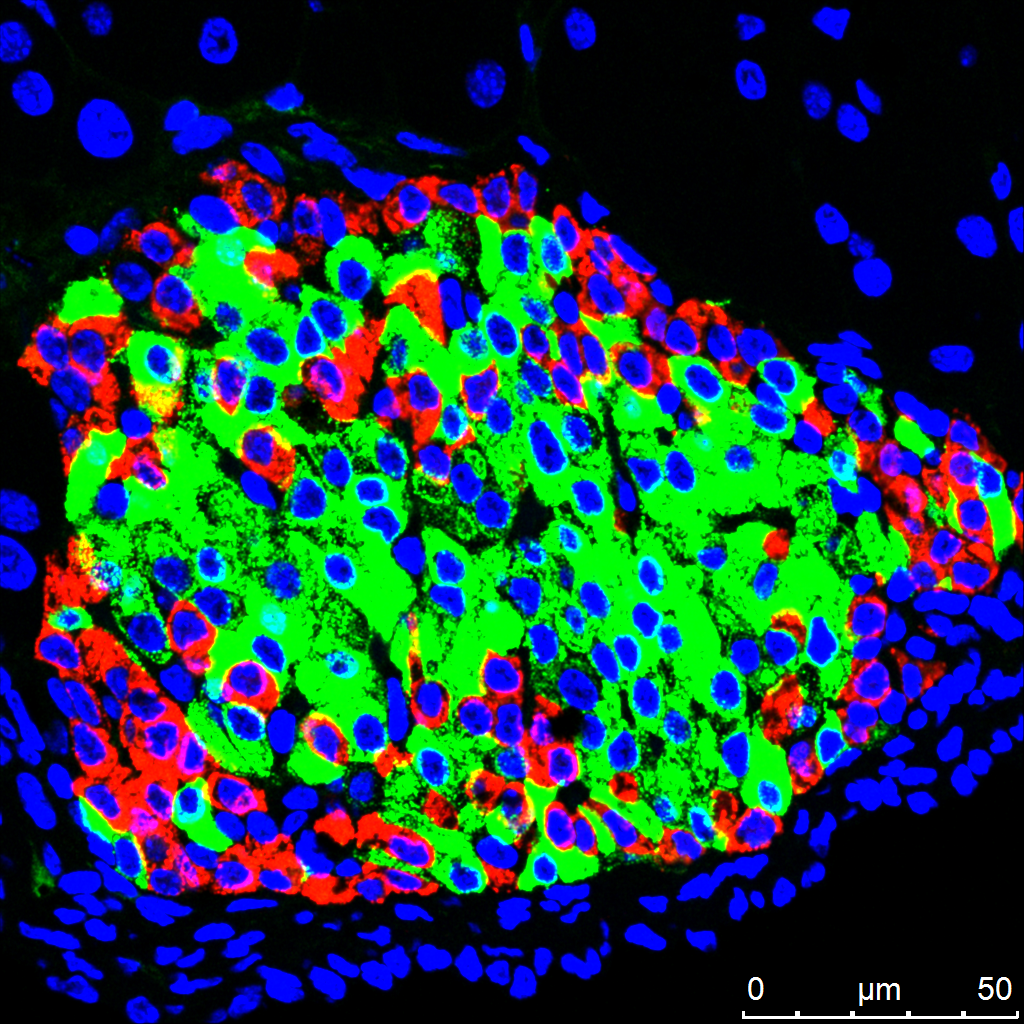

Supplement: S1 File — (ZIP) [file pone.0260100.s001.zip › PONE-D-21-15861R1-minimal data set-20211205yf/PONE-D-21-15861R1-Fig 1c-1d/Fig1c STZ.tif]

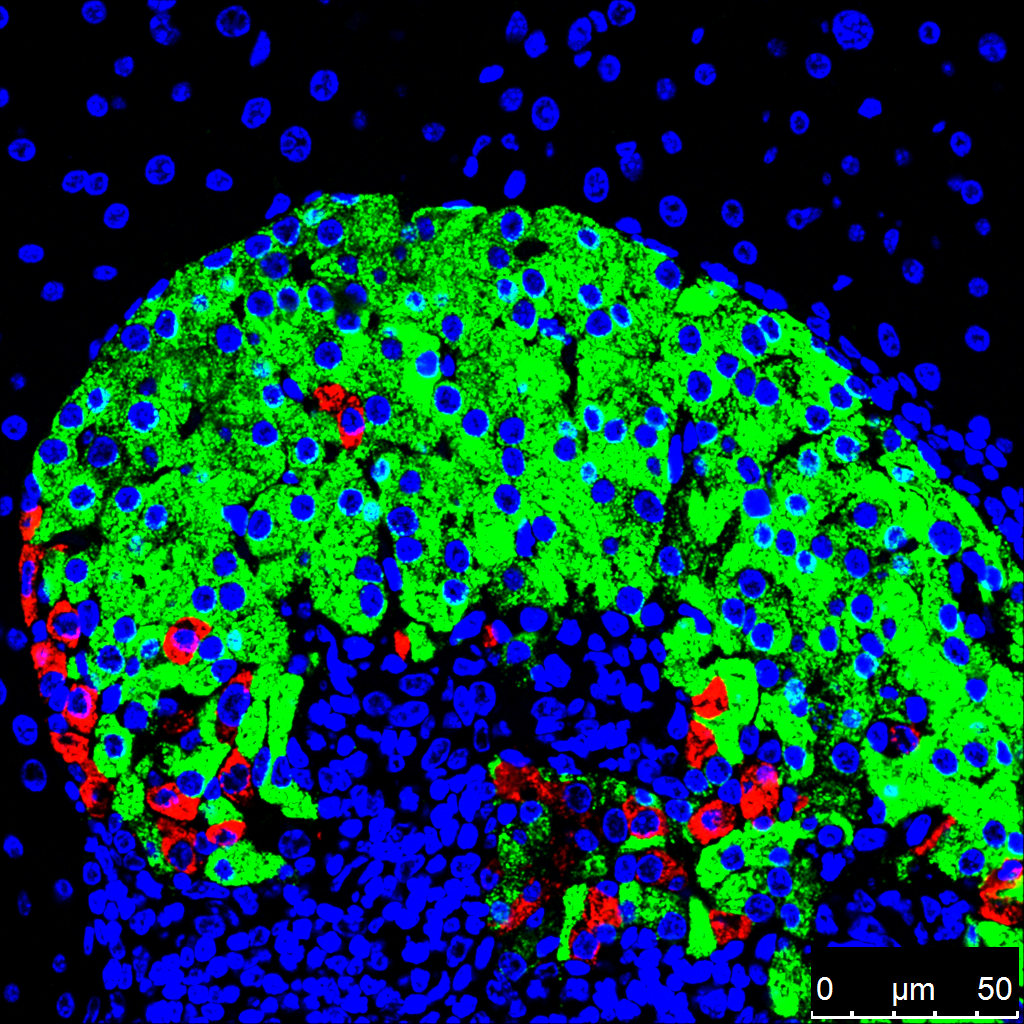

Supplement: S1 File — (ZIP) [file pone.0260100.s001.zip › PONE-D-21-15861R1-minimal data set-20211205yf/PONE-D-21-15861R1-Fig 1c-1d/Fig1d NOD(10w).tif]

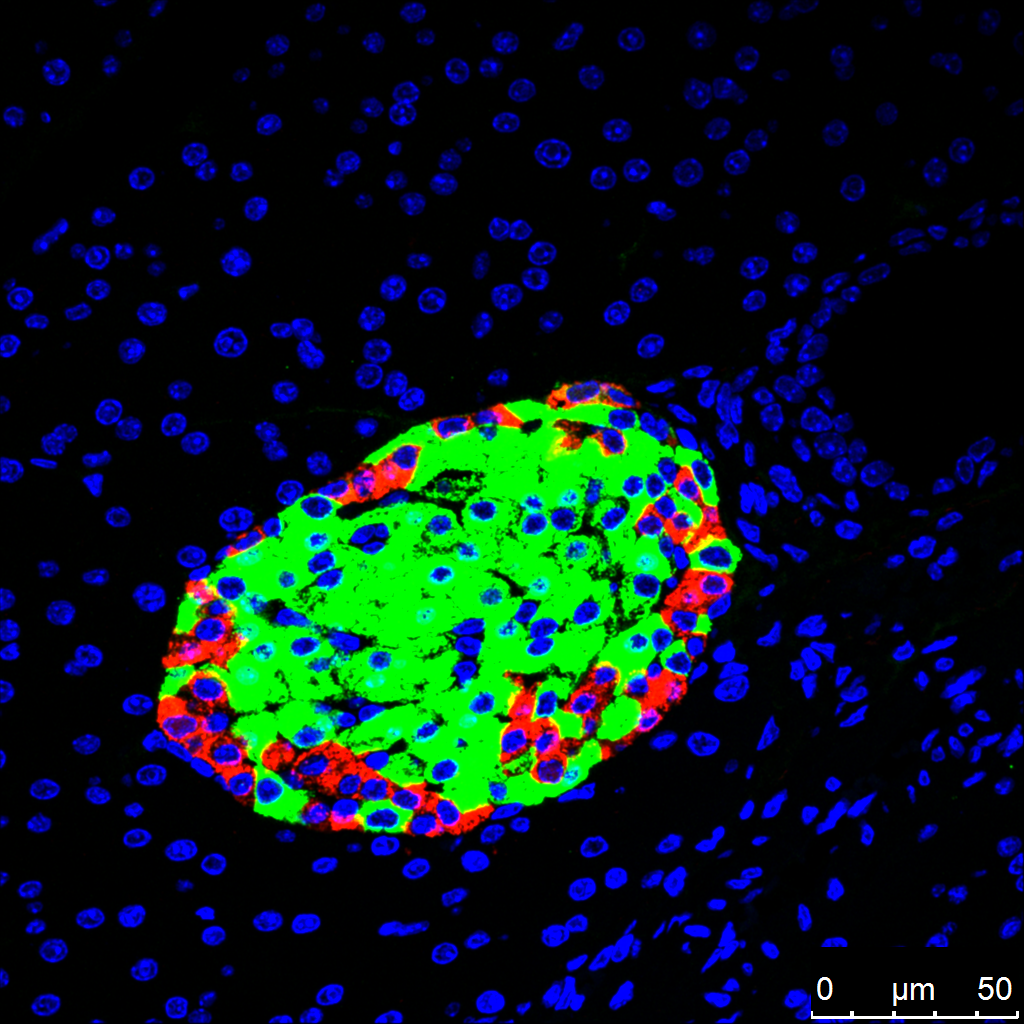

Supplement: S1 File — (ZIP) [file pone.0260100.s001.zip › PONE-D-21-15861R1-minimal data set-20211205yf/PONE-D-21-15861R1-Fig 1c-1d/Fig1d NOD(2w).tif]

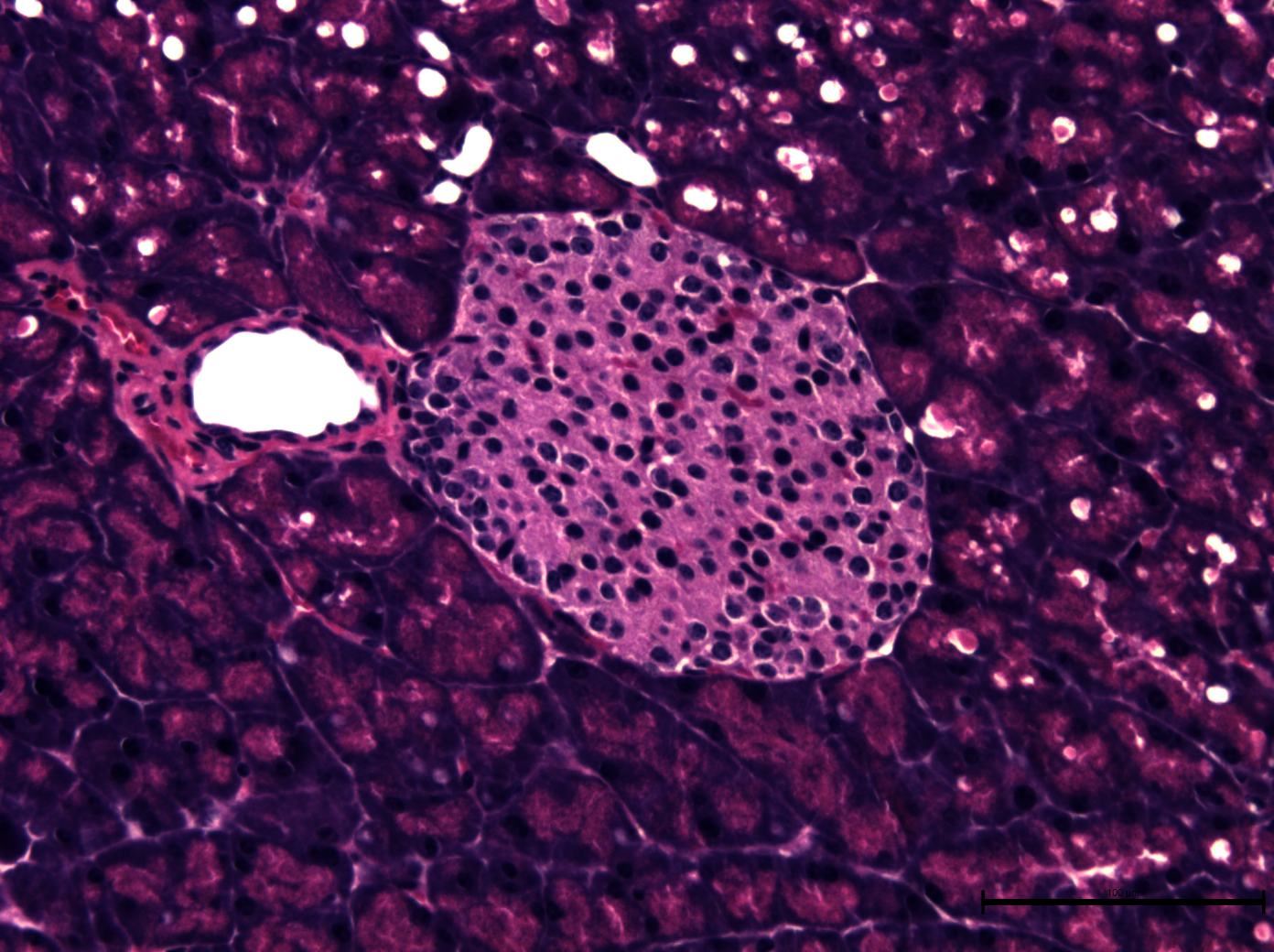

Supplement: S1 File — (ZIP) [file pone.0260100.s001.zip › PONE-D-21-15861R1-minimal data set-20211205yf/PONE-D-21-15861R1-Fig 3a-3c/Fig3a DT.tif]

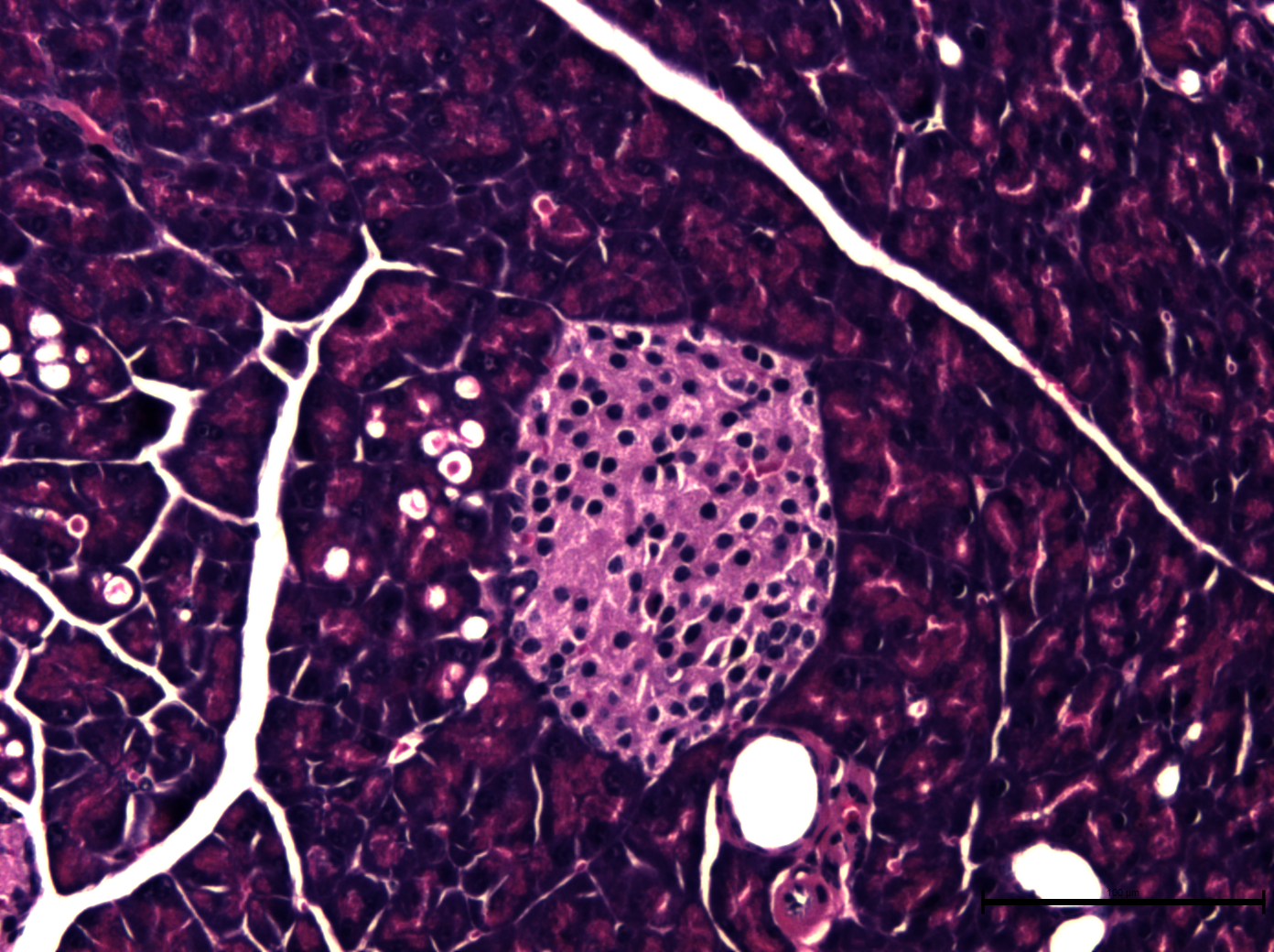

Supplement: S1 File — (ZIP) [file pone.0260100.s001.zip › PONE-D-21-15861R1-minimal data set-20211205yf/PONE-D-21-15861R1-Fig 3a-3c/Fig3a PBS.tif]

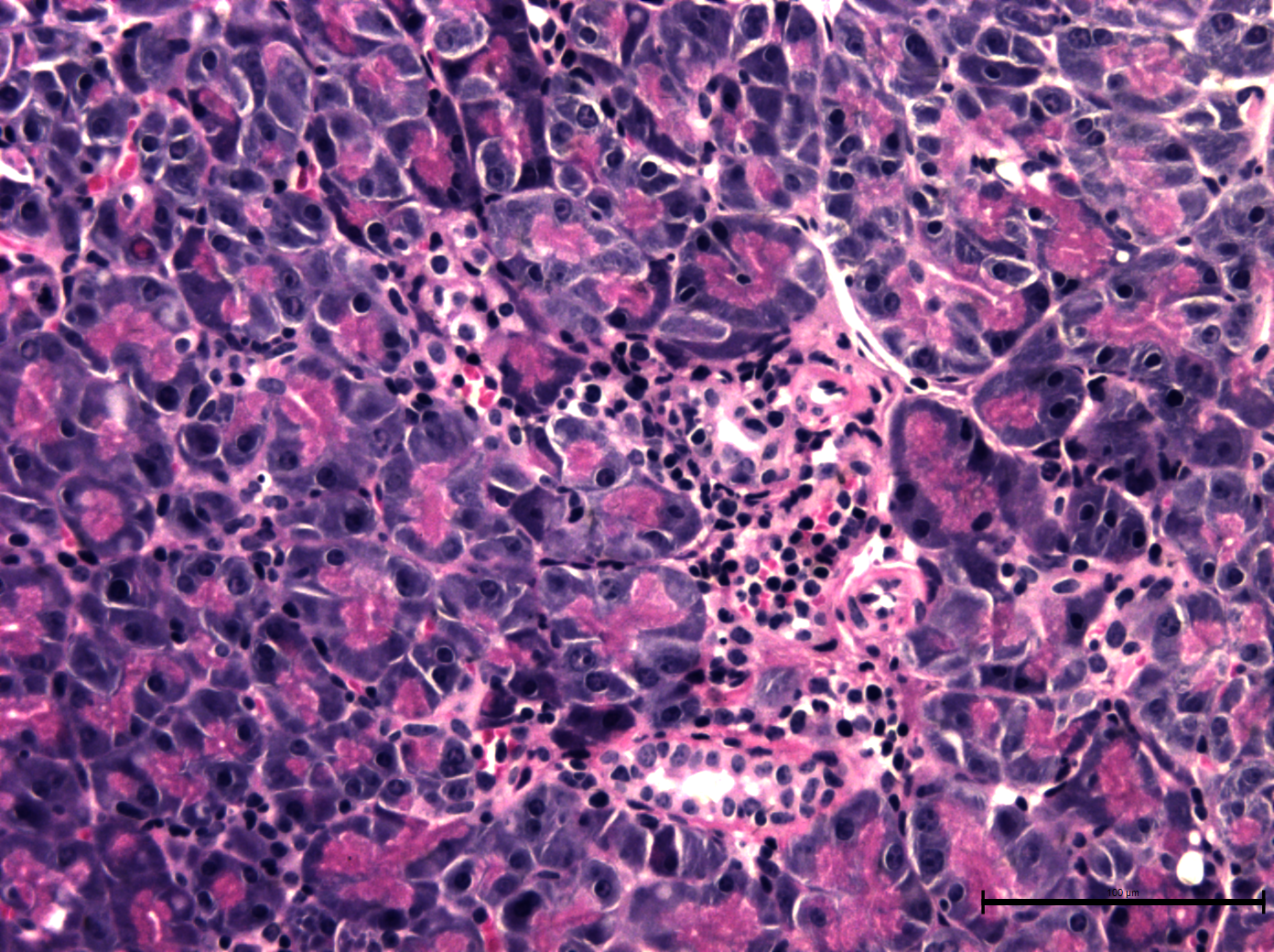

Supplement: S1 File — (ZIP) [file pone.0260100.s001.zip › PONE-D-21-15861R1-minimal data set-20211205yf/PONE-D-21-15861R1-Fig 3a-3c/Fig3a STZ+DT.tif]

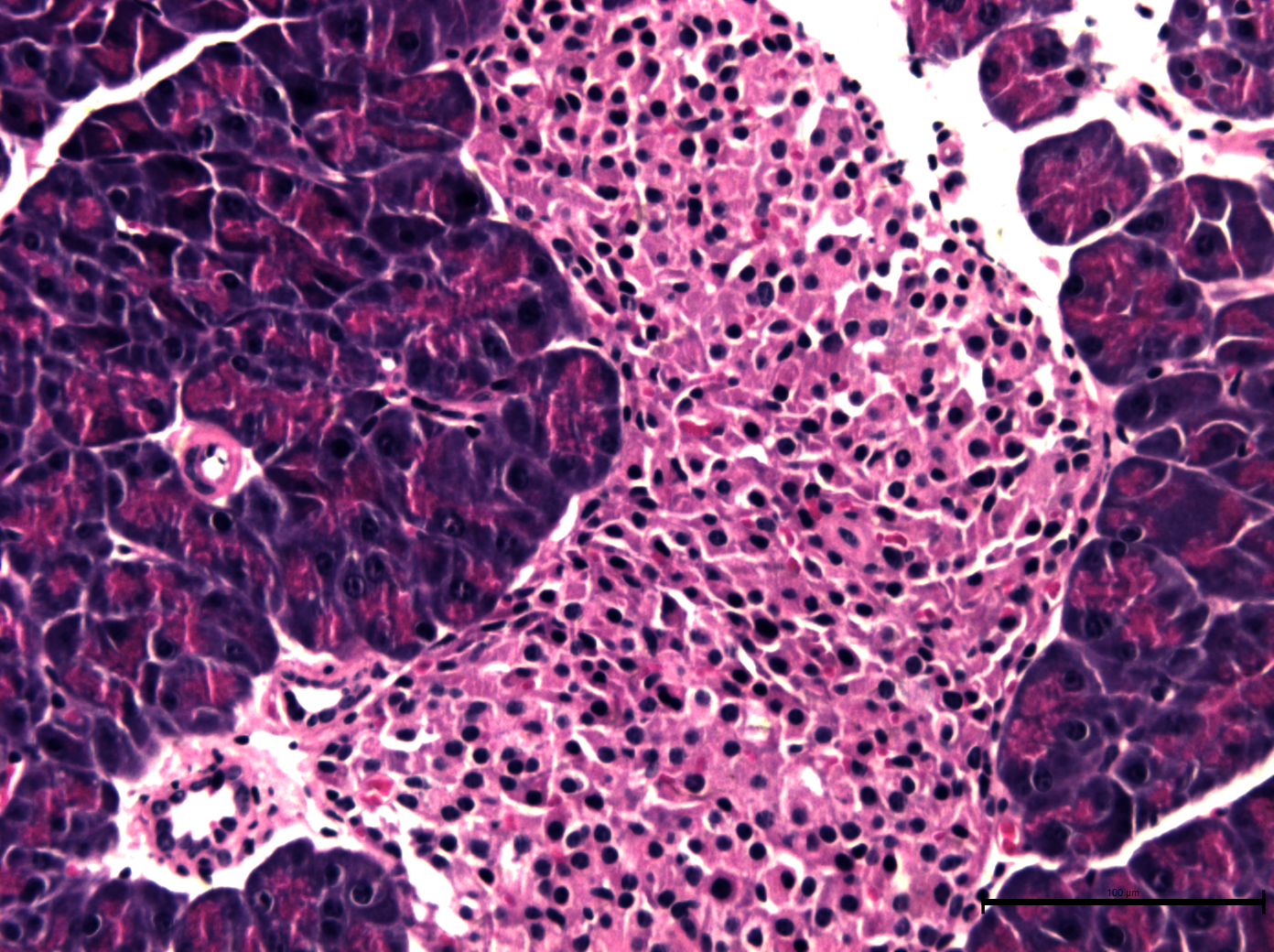

Supplement: S1 File — (ZIP) [file pone.0260100.s001.zip › PONE-D-21-15861R1-minimal data set-20211205yf/PONE-D-21-15861R1-Fig 3a-3c/Fig3a STZ.tif]

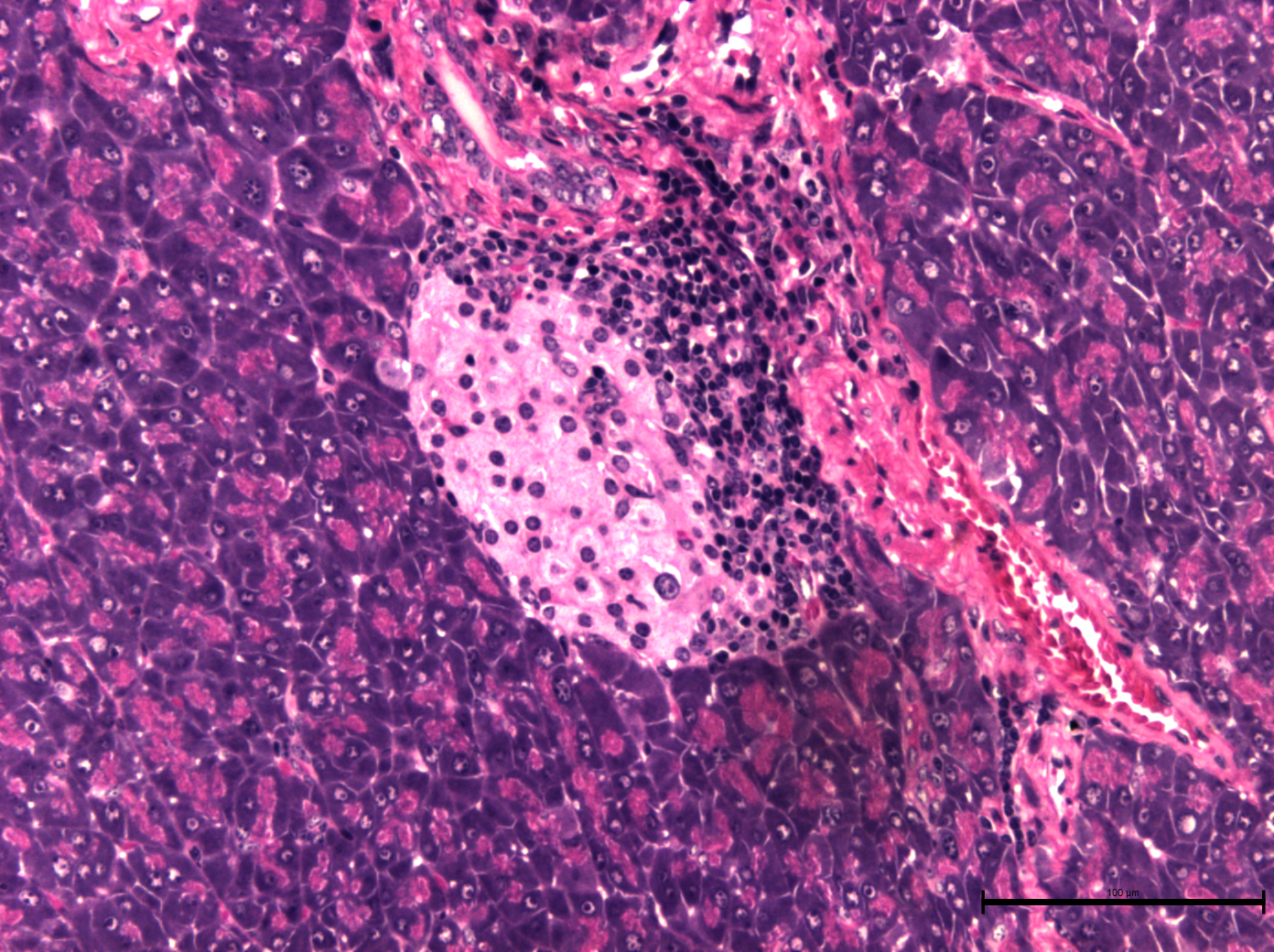

Supplement: S1 File — (ZIP) [file pone.0260100.s001.zip › PONE-D-21-15861R1-minimal data set-20211205yf/PONE-D-21-15861R1-Fig 3a-3c/Fig3b NOD(10w).tif]

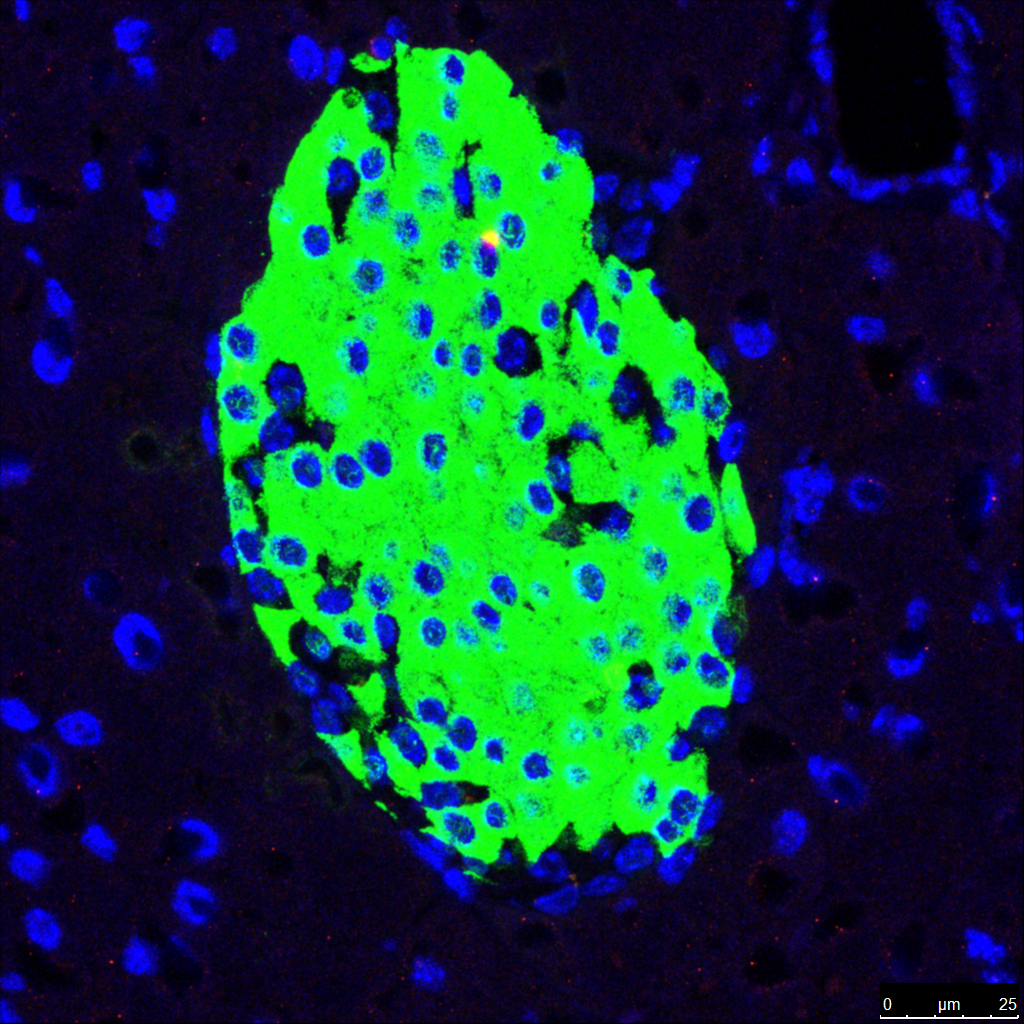

Supplement: S1 File — (ZIP) [file pone.0260100.s001.zip › PONE-D-21-15861R1-minimal data set-20211205yf/PONE-D-21-15861R1-Fig 3a-3c/Fig3c DT.tif]

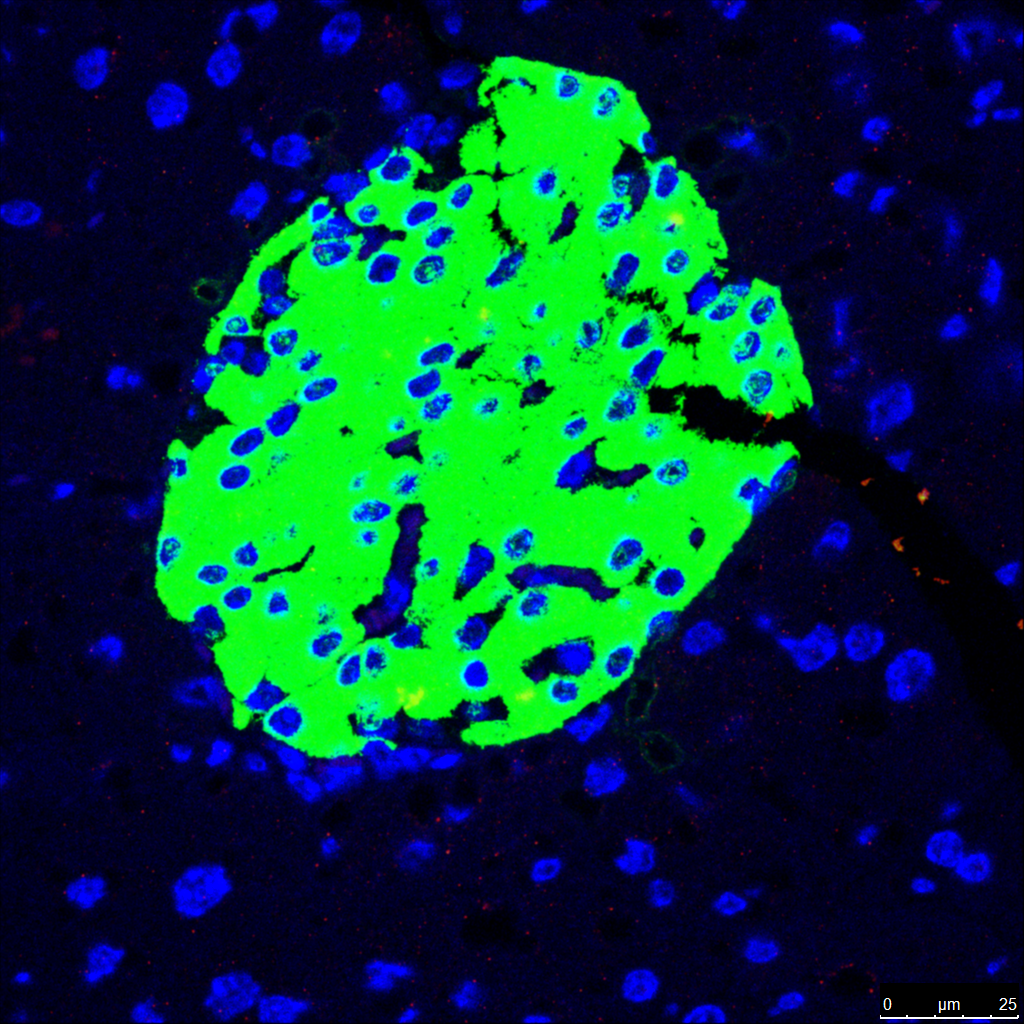

Supplement: S1 File — (ZIP) [file pone.0260100.s001.zip › PONE-D-21-15861R1-minimal data set-20211205yf/PONE-D-21-15861R1-Fig 3a-3c/Fig3c PBS.tif]

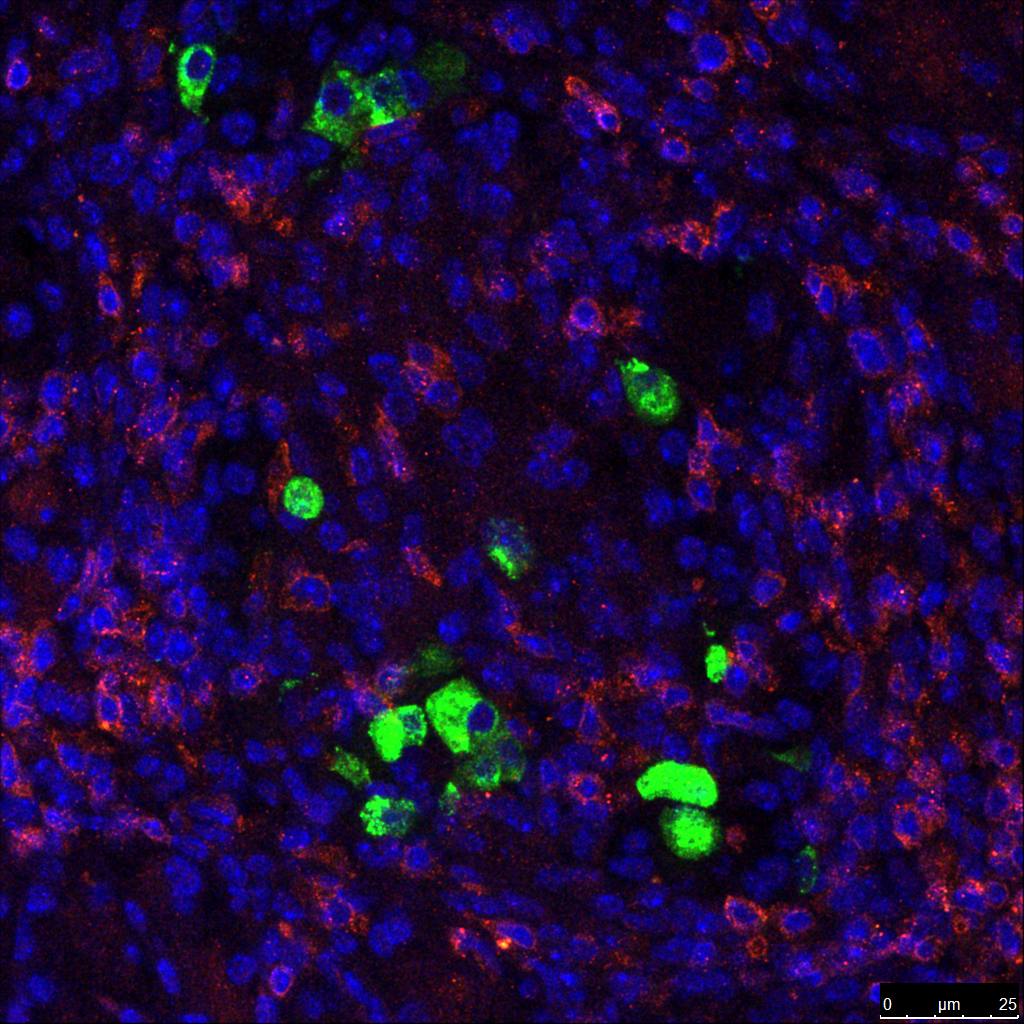

Supplement: S1 File — (ZIP) [file pone.0260100.s001.zip › PONE-D-21-15861R1-minimal data set-20211205yf/PONE-D-21-15861R1-Fig 3a-3c/Fig3c STZ+DT.tif]

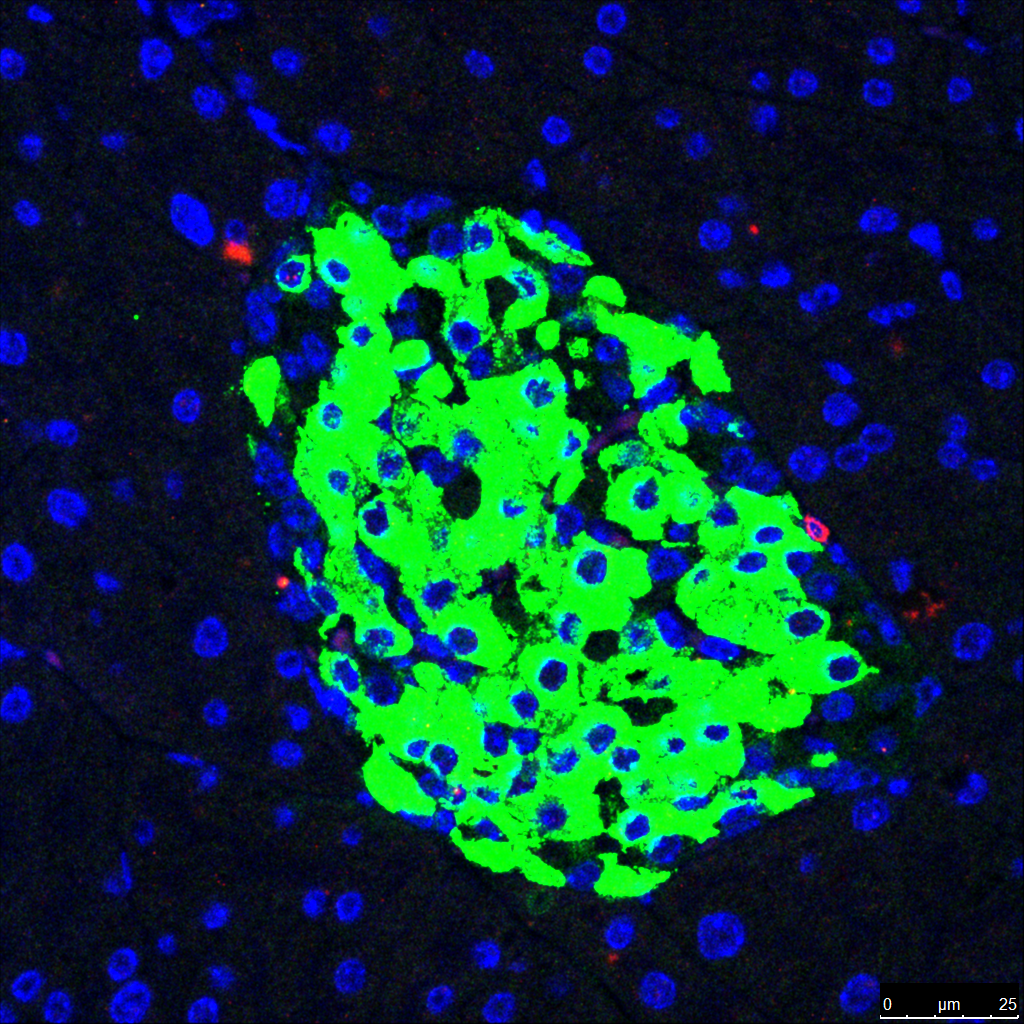

Supplement: S1 File — (ZIP) [file pone.0260100.s001.zip › PONE-D-21-15861R1-minimal data set-20211205yf/PONE-D-21-15861R1-Fig 3a-3c/Fig3c STZ.tif]
