# Supplementary material for: Rational construction of controllable autoimmune diabetes model depicting clinical features
Source: PLoS One. 2022 Jan 21;17(1):e0260100. doi: 10.1371/journal.pone.0260100 (PMC8782301; doi:10.1371/journal.pone.0260100)
Supplement: Attachment — Submitted filename: Response to Reviewers.docx [file pone.0260100.s002.docx]

Response to Academic Editor and reviewers’ Comments

**Point 1:** ****Request from staff editors****

We note that your study design may include death of a regulated animal as a likely outcome or planned experimental endpoint. At this time, we request that you please report additional details in your Methods section regarding animal care and use for the survival study, as per our editorial guidelines (http://journals.plos.org/plosone/s/submission-guidelines#loc-humane-endpoints).

**Response 1**: Thanks for your request, we have added the paragraph to describe animal care and use for the survival study in our Methods section, from line 198 to line 208, including description of the humane endpoints in this study.

**Point 2:** Journal Requirements:

Please review your reference list to ensure that it is complete and correct. If you have cited papers that have been retracted, please include the rationale for doing so in the manuscript text, or remove these references and replace them with relevant current references. Any changes to the reference list should be mentioned in the rebuttal letter that accompanies your revised manuscript. If you need to cite a retracted article, indicate the article’s retracted status in the References list and also include a citation and full reference for the retraction notice.

**Response 2:** Thank you for the suggestion**.** I have checked the whole reference listed online, no any article cited was retracted. No necessary change for the current reference list.

**Reviewers' comments:**

**We did not receive any review comments yet.**
